# Individual spindle detection and analysis in high-density recordings across the night and in thalamic stroke

**DOI:** 10.1038/s41598-018-36327-x

**Published:** 2018-12-14

**Authors:** A. Mensen, R. Poryazova, R. Huber, C. L. Bassetti

**Affiliations:** 1 Department of Neurology, University Hospital (Inselspital) and University of Bern, Bern, Switzerland; 2 Giga-Consciousness, Coma Science Group, University and University Hospital of Liege, Liege, Belgium; 3 Department of Neurology, University Hospital Zurich, Zurich, Switzerland; 4 Child Development Center, University Children’s Hospital Zurich, Zurich, Switzerland

## Abstract

Sleep spindles are thalamocortical oscillations associated with several behavioural and clinical phenomena. In clinical populations, spindle activity has been shown to be reduced in schizophrenia, as well as after thalamic stroke. Automatic spindle detection algorithms present the only feasible way to systematically examine individual spindle characteristics. We took an established algorithm for spindle detection, and adapted it to high-density EEG sleep recordings. To illustrate the detection and analysis procedure, we examined how spindle characteristics changed across the night and introduced a linear mixed model approach applied to individual spindles in adults (n = 9). Next we examined spindle characteristics between a group of paramedian thalamic stroke patients (n = 9) and matched controls. We found a high spindle incidence rate and that, from early to late in the night, individual spindle power increased with the duration and globality of spindles; despite decreases in spindle incidence and peak-to-peak amplitude. In stroke patients, we found that only left-sided damage reduced individual spindle power. Furthermore, reduction was specific to posterior/fast spindles. Altogether, we demonstrate how state-of-the-art spindle detection techniques, applied to high-density recordings, and analysed using advanced statistical approaches can yield novel insights into how both normal and pathological circumstances affect sleep.

## Introduction

Sleep spindles are among the most recognisable electrophysiological features occurring in non-rapid-eye-movement (NREM) sleep. They can be readily seen in continuous electroencephalography (EEG) traces as a series of stereotypical sinusoidal waves waxing and waning at frequencies between 11–15 Hz for approximately a second^1^. Distinct spindle properties have been associated with a host of behavioural and clinical phenomena^2^. The most closely studied link between spindles and cognition has been to memory and learning, in particular the role of spindles in sleep memory consolidation and plasticity^3–5^. While most of the associations are correlational, recently it was shown that specific enhancement of spontaneous spindles using transcranial electric stimulation, could improve memory performance compared to sham^6^. At the clinical level, both reductions in spindle power and incidence have been reported for schizophrenia patients^7,8^. Furthermore, damage to certain parts of the thalamus, particularly after stroke, have also been associated with a reduction in spindle activity^9,10^. It may well be that both the spindle changes in schizophrenia and after thalamic damage are closely related^11^. Thalamic activity, specifically, has been consistently associated with spindling activity in both human^12,13^ and animal studies^14^.

Much of the research on sleep spindles is based on measuring spectral power in the spindle range of frequencies during NREM. Spectral power has been a useful tool in capturing aspects of spindles which differ between experimental conditions and/or distinct populations. However, it has its limitations. No specific standard exists for spectral calculation parameters and the assumption of stationarity underlying power calculations is unfounded as spindles are relatively rare and spontaneous events. Changes in spindle power may be due to changes in the incidence of spindles, amplitude, duration, topographical spread, or shifts in frequency. Thus if spectral power shows significant differences, it is important to further investigate which particular spindle property was most affected. With the advent of increasing computational power and advanced algorithms we can detect and examine individual spindle properties at virtually no additional costs compared to the traditional calculation of spectral power.

Several spindle detection algorithms have already been suggested, and their performance has been compared and reviewed to a gold standard of expert scorers^15^. While single channel comparisons to experts is undoubtedly useful, manual detection is practically impossible for multi-channel recordings for whole night recordings over multiple participants. The current paper has two principle aims. While the homeostatic elements of slow waves have been explored for decades^16–18^, changes in spindle properties over the course of the night have been relatively unexplored^19^. As such, we used this opportunity to outline and demonstrate the tools required for automatic spindle detection and analysis, with specific focus on how previous tools can be extended for multi-channel EEG recordings, by examining how individual spindle properties change through the course of a night of sleep. The second is to show how spindles can, and should, be analysed at the individual level using a mixed model approach in both a healthy population, as well as a clinically interesting, set of patients following thalamic stroke.

## Methods

### Datasets

Two distinct datasets were used. A set of 9, relatively young, all male, controls (age range: 25–35), were randomly selected from the Wisconsin Sleep Cohort with no previous history of sleep disorders or any other signs of neurological impairment^20^. The same set of participants were previously used to examine the detection and analysis of slow waves^21^ (two participants were excluded since there was no consistent late cycle of NREM sleep). An elderly control and thalamic stroke group (*n* of 9 for each group) were part of a previous research study examining the effect of stroke on slow-wave-activity^22^; however none of the thalamic stroke patients were previously published. The elderly and stroke group were matched for gender (5 males) and age (mean = 62.9 for both groups; +−12.5 and +−11.7 respectively). Patients were measured a mean of 7.8 days (+−4.8) after their stroke. Six patients had unilateral damage (3 L/3 R), and 3 had bilateral damage. All studies had been approved by their respective local ethics commissions (Health Sciences Institutional Review Board of the University of Wisconsin–Madison and the Cantonal Ethics Commission of Zurich). All participants signed written informed consent forms for their participation and further analysis of their datasets anonymously. This research was conducted in accordance with Good Clinical Practice guidelines and regulations.

Recordings in both datasets were performed using the EGI NA300 amplifier sampling at 500 Hz, and the EGI geodesic *net*^23^. Sleep was scored according to the AASM criteria by selecting the sensor-net equivalents for the standard channels (REOG, LEOG, F3, F4, C3, C4, O1, and O2), and referencing them to the contralateral mastoid channel. Two posterior lateral electrodes referenced to one another served as the EMG channel for muscle tone. The timing of early and late NREM cycles was determined by consecutive epochs of stage 2 or 3 with a minimum of 30 minutes not interrupted by a single *wake* epoch and no more than one epoch of stage 1. Bad channels were manually detected and interpolated using spline interpolation^24^, (young controls mean number of channels = 7.1, std = 4.8; elderly and stroke population = 1.8 std = 2.0). Signals were then referenced to the average activity over all channels. Interpolation was done prior to average referencing to avoid any potential lateralisation bias from the artefactual channels, and to keep the degrees of freedom for channel level calculations consistent at the group level. Technical artefacts in these sleep episodes were manually marked and excluded from the spindle detection. The datasets generated during and/or analysed during the current study are available from the corresponding author on reasonable request.

### Spindle Detection

Spindle detection, analysis and visualisation were performed using the *sleep wave analysis* toolbox; an open-source toolbox^21^ (github.com/Sinergia-BMZ/swa-matlab). Detection proceeded along three key stages. First, the average activity of small regions along the scalp was calculated. These *canonical* time-series act to initially reduce the channels on which spindle detection runs while also improving signal-to-noise ratio of the spindle events. Since spindles tend to be relatively local events^25,26^, the current analyses used the *‘grid’* regions option which examined the activity from 9 non-overlapping regions: 3 frontal, 3 central, and 3 posterior. The regions were equidistant from each other, non-overlapping, and contained an average of 7 channels (std = 2.7) each.

The second stage examined the canonical time-series for spindles along similar steps to that of Wamsley *et al*.^8^ (further reviewed in^15^). First, a wavelet-based filter was performed on each of the canonical time-series using a *b-spline* wavelet^27^. The power of the resulting signal was measured by squaring the values and smoothing the time-series using a sliding window of 100 ms. An initial *high-*threshold was then applied, looking for power values that were at least 4 times the median absolute deviation. Events crossing this *high-*threshold were considered potential spindles whose start and ends were then measured using the crossing time at a second, *low-*threshold at 2 median absolute deviations^25,28^. For the analyses presented here, these thresholds were determined for each cycle separately, however researchers may also choose to use a single threshold when comparing sleep across the same individual as in the first dataset. Valid spindles durations were, at least, 300 ms to maximum 3 seconds. These criteria were applied independently for each canonical region. Any overlap in timing (i.e. start to end), between spindles of distinct canonical regions were considered as single events and their maximal parameters combined (ie spindle duration could have its start in one canonical time series and its end in another; largest power among canonical regions taken as the *summary* value for that spindle).

**Figure 1:**
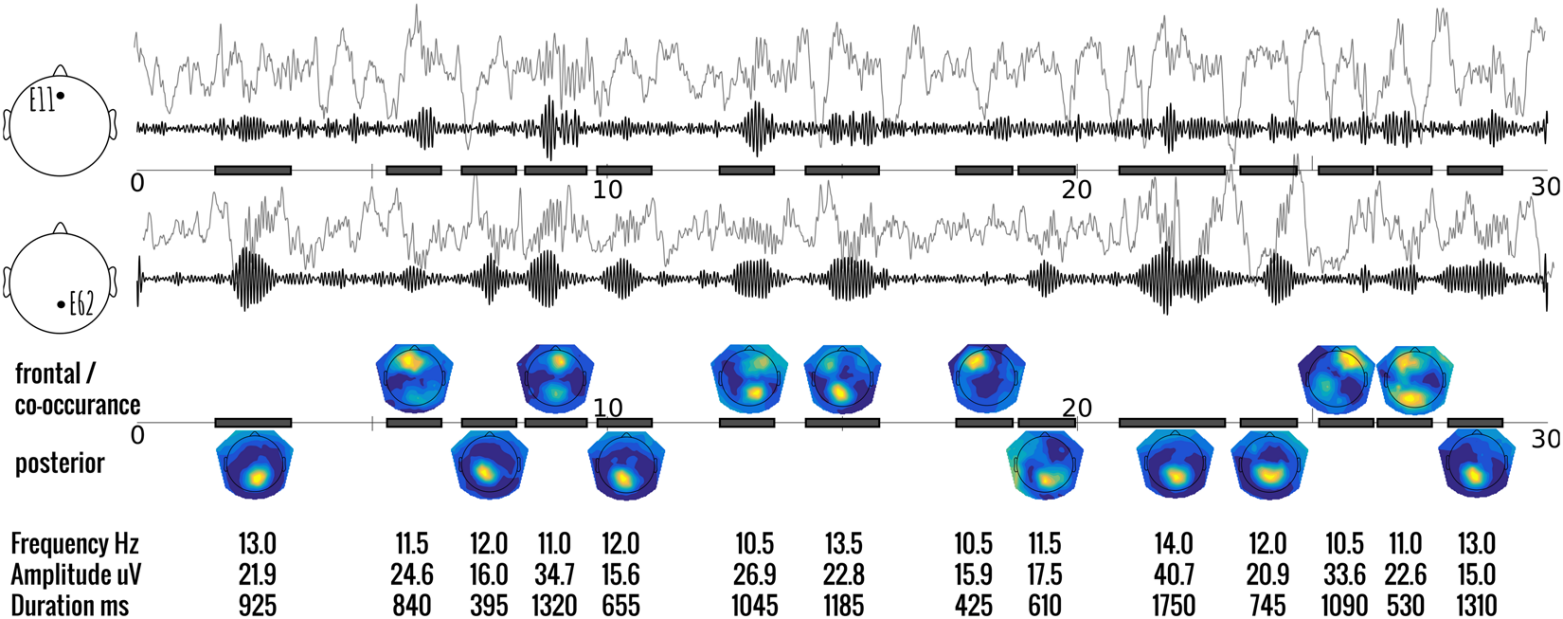
Representative trace of multiple spindle events during 30 seconds of NREM sleep. The top 2 panels show the typical ‘raw’ EEG signal (elevated grey) and the wavelet filtered (10–16 Hz) signal (black) of a selected frontal-central channel (E11), and posterior-central channel (E62) where spindles are most typically found. Detected spindles are indicated with a black bar in the timeline. Keep in mind that the detected spindle may not have been found in either E11, or E62 (e.g. 8^th^ and 12^th^ example spindle. The third panel shows the topography of the mean spectral power over all detected spindles. Locations tend to be either frontal or posterior, with fewer occurring at both locations simultaneously. The bottom panel shows some of the individual parameters that can be assessed from the results of this automatic detection.

Once all the potential spindles had been detected in the canonical series, the original channels were re-examined. Here, spectral power at each channel, during the time period of the spindle event, was calculated using the pwelch method. Three key parameters are obtained here: the power at each channel; the peak frequency; and the ratio of the mean power in the defined spindle range over the mean power in the neighbouring ranges (8–10 Hz and 16–18 Hz). *Power*-*ratio* ensures some specificity of the transient power increases within the spindle range. For example, short arousals may involve more broadband increases in power which include the spindle range. While some previous work has recognised the problem of broadband increases^26^, the use of a power-ratio is, to the best of our knowledge, a novel criteria. An important distinction to keep in mind is that this measure of power is over the individual spindle, and not over the entire N2 or N3 period as most measures of spindle power do; hence stationarity can be assumed. A spindle was considered to be active at the single channel level if this power ratio was above an adjustable threshold; set to 1.5 in our case. In other words, the power in the defined spindle range needed to be more than 50% stronger than in the neighbouring ranges to be detected at the individual channel level. Furthermore, we could categorise each spindle by their topographic location, ie *spindle-type, as*: *posterior* if the power at the posterior canonical region is at least 50% larger than the frontal region, and vice-versa (see Fig. 1 for individual examples). In the case where the power did not exceed this arbitrary 50% cutoff, the spindle-type was considered to be *co-occuring*. While the pwelch method employing the fast fourier transform is a common measure, it is used here at the individual spindle level where the total window length may be quite short. Therefore, there may be alternative measures of power that could be used as well (e.g. using the peak of the smoothed wavelet coefficient as calculated at the canonical level).

There is still considerable disagreement about optimal spindle detection parameters (see a recent editorial on the topic^29^). We emphasise that the specifics of the methodology or individual parameter criteria used are not of primary concern here as long as individual parameters are assessed and given as an output. While we are confident that our parameter settings are generally robust, and we could not find clearly better parameters, without a proper gold standard we cannot go as far as stating that they are the most optimal possible. One internal check that can be made is to compare the power spectra between the data with and without the detected spindles (see Supplementary Fig. 1). Reasonable parameter settings should reduce the classic spindle bump between 10 and 16 Hz, without reducing the power in other bands. Within these reasonable boundaries we can expect a trade-off between sensitivity and specificity. We recommend using settings in the more sensitive range (e.g. broad duration criteria, relatively low thresholds), since the individual-spindle analysis approach used here (detailed below) can explore any interaction between conditions of interest, and specific spindle parameters. Such an approach can mitigate the effects of detection criteria differences since these could be accounted for at the analysis stage.

### Spindle Analysis

Detection results in a large table with specific information about each spindle. Several measurements can be directly compared or derived to examine the individual, condition, or group differences. Here, we focus on a subset of these spindle properties. Spindle *incidence* was defined as the number of unique spindle detected, per minute of artefact-free N2/N3. Here we can distinguish between *total spindle incidence* as the rate of detecting a spindle at any position on the scalp, as well as channel-level incidence which indicates whether the spindle event identified in any of the canonical regions was present at each particular channel. Spindle *globality* described the number of channels in which a particular spindle was detected (at the canonical level) as a percentage of total channels recorded. Spindle *amplitude* reflected the largest peak to peak difference in a canonical region. Spindle *duration* was defined as the time between an individual spindle’s first appearance in a canonical time series to the last. Spindle *power* and *peak frequency* were calculated using a fast fourier transform spectral analysis over this spindle duration. *Peak frequency* was measured for each of the canonical regions across the midline independently as this is a well known property to vary across topography^30^.

### Statistical Analyses

All analyses were performed with Matlab 2017a (Mathworks, Natick, 2017), using custom scripts and the *Statistics and Machine Learning* toolbox. The first dataset was first analysed using traditional pairwise (i.e. dependent) t-tests on the *means* of each spindle parameter of interest across the conditions (*time of night*; early versus late). We can see from Fig. 1 that the parameters of individual spindles are relatively robust and the *participant mean* cannot capture the apparent relationships between parameters (e.g. lower frequency peaks of frontal dominant spindles). Nor can we be confident whether this mean value is a good representative (see Supplementary Fig. 2 for data distribution). As Cox *et al*.^30^ demonstrated, individual variability in spindle dynamics is masked by group-level analysis and we should take advantage, “rather than ignore” this by examining differences at the individual spindle level. Since spindles from the same participant cannot be seen as independent measures we cannot run a linear regression. We also cannot perform a repeated measure ANOVA because we will necessarily have an imbalance in the number of spindle types, along with several continuous covariates.

Linear mixed models (LMM) are ideally suited for this sort of data structure and here we introduce the concept to perform the statistical analysis at the individual spindle level. We treat each participant as a *random effect* to deal with the expected correlations among the distinct spindles from each participant. Apart from its statistical validity, the LMM approach inherently weighs the analysis for the number of spindles found in each recording. Secondly, continuous covariates to the principal parameter of interest can be included to account for variance in the measure that would have been considered as error otherwise.

Model specification and comparison is also a key feature of the LMM approach^31^. However, it is unfeasible to examine all potential models as this quickly becomes and *NP-hard* problem with the large number of potential predictors available here. Yet, a narrow focus on hypothesis driven models may miss out on novel aspects which could play a crucial role in the interpretation and consequences of the analysis. Our proposed strategy is to separate the list of potential predictors into 2 categories: *key predictors* and *potential covariates*. To eliminate any collinearity from the model, we first modelled each potential covariate using the key predictors and used the residuals of these models as the new covariate values. We then used a stepwise regression algorithm (Matlab’s *stepwiselm*) to examine which combination of covariates would best explain our dependent variable of interest. The full interaction of key predictors served as the starting condition and then each potential covariate was added to the model and examined.

There is, currently, no openly available algorithm for stepwise model specification optimisation for mixed models (or other optimisations; e.g. LASSO). Therefore, since the mixed model approach may have yielded distinct predictors, we compared the predictor lists generated using distinct optimisation parameters for the stepwise regression, using Likelihood Ratio tests of the subsequent mixed model with those optimisers^32^. We found that using the AIC optimiser in the linear stepwise regression consistently produced the *best* model for the linear mixed model approach. Moreover, this approach generally converged to the same set of predictors even when changing the starting conditions of the key predictors (e.g. additive versus interaction models). Once the *best* model has been derived, the main effect of key predictors is calculated by comparing this *best* model to a reduced model without the particular term of interest, using the likelihood ratio test.

### Topographic Analysis

The LMM approach, as currently defined, is reliable approach to examining the spindle summary measures. However, if applied at the single channel level independently, we are faced with the classic multiple-comparisons-problem of determining statistical significance in a group of semi-independent tests. We therefore also extended this LMM approach to the analysis of individual channels using a method from both EEG and fMRI statistical analysis specifically designed to handle this issue: threshold-free cluster-enhancement (TFCE), followed by a maximum permutation approach^33^ (https://github.com/Mensen/ept_TFCE-matlab). For the analysis of the second dataset, we first calculated the LMM for a single channel. The observed T-values for each term in the model were then stored. Next, a new data table was created by randomising the values for each specific factor (i.e. *key predictor*) of interest. For this analysis of the second, stroke dataset, we randomised both the *spindle type* (a within-subjects factor) and *stroke location* (a between-subjects factor). For between-subjects factors, the values are randomised such that all the individual spindles for a single participant are relabeled together. For within-subjects permutations, values are relabeled for each participant independently such that the total number of each level are maintained at the participant level. The same LMM is then run on this new dataset and the T-values for each term in the model is stored. For the current analysis, 2500 randomised datasets were created and examined. Once this process is completed for each channel independently, the statistical values from both the observed and permuted models are then subjected to the TFCE approach which examines each channels’ support from its neighbours and adjusts the values accordingly.

To control for multiple comparisons, a single empirical distribution is created using the maximum value from each of the permuted datasets, irrespective of the particular channel this value comes from. Statistical significance for each channel is determined by counting the proportion of statistical values in the empirical distribution that are greater than the original test statistic for that channel^34,35^. See Supplementary Fig. 3 for a comparison of the significance levels of the TFCE approach compared to the max permutation approach which does not account for channel neighbourhood. A further advantage to the permutation approach is that it takes the structure of the design matrix into account while determining statistical significance. In doing so, it can minimise the impact of over-fitting in the LMM with a large number of predictors.

## Results

### Early versus late night spindles

**Figure 2:**
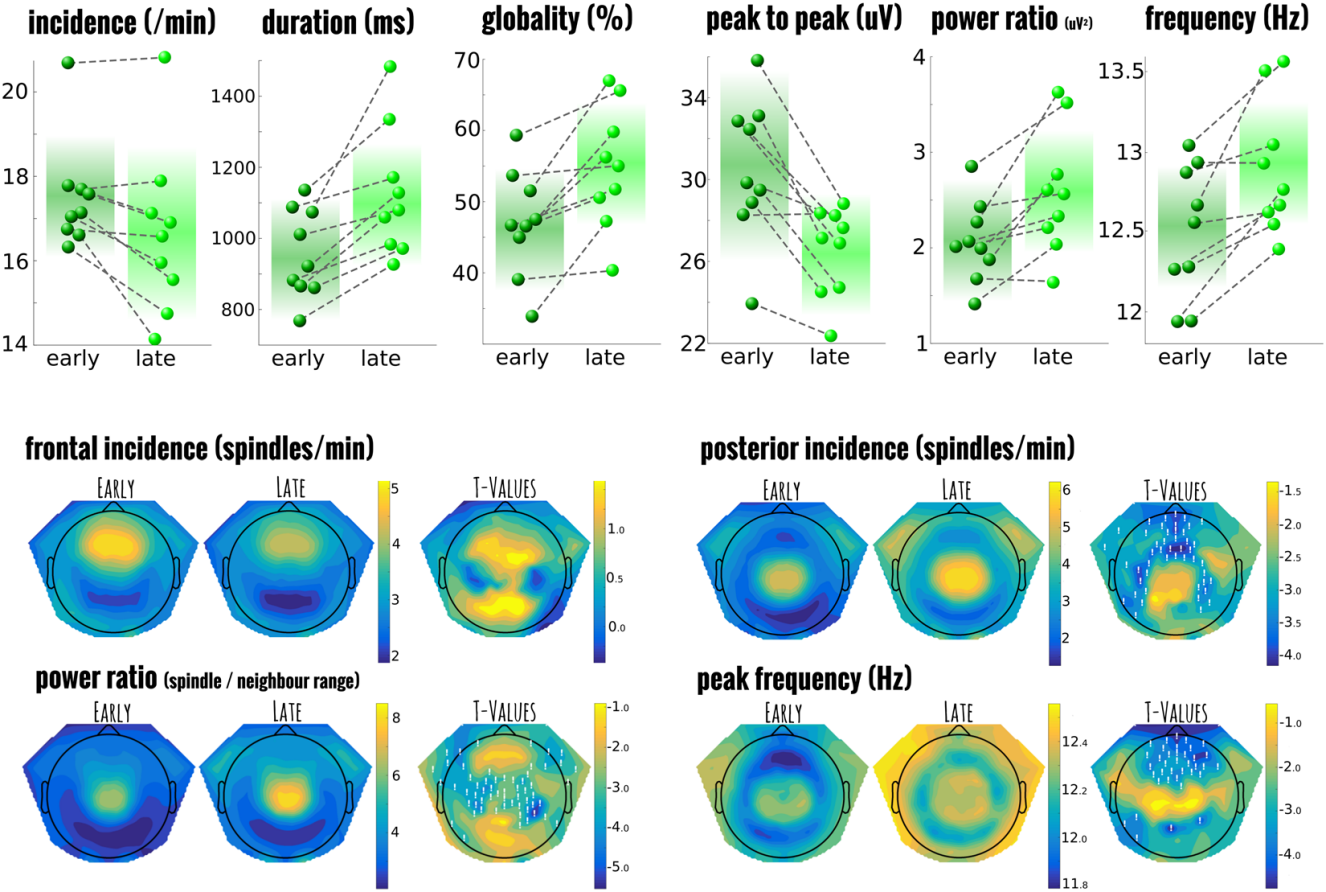
Comparison between early night and late night spindles in healthy controls. Top panel shows the individual participant values for each of the selected spindle summary measures and how they change over the course of the night from the first to last cycle of sleep. The background colour indicates the mean value with fade out at 2 standard deviations. Notice that while the power of spindles (in ratio of spindle frequencies to its neighbours) increases across the night, the amplitude and incidence of individual spindles actually decreases while the duration and globality increase. All comparisons are significantly different from one another. The panels below the t-value topography of selected spindle measures and their statistical comparison; channels marked with a white ‘!’ are statistical different after correction for multiple comparisons.

Figure 2 summarises the selected measures and group comparisons comparing early (first N2/3 cycle of sleep), to late sleep (typically 4th or 5th cycle of N2/3). Table 1 shows the estimated differences, and corresponding statistical comparisons between each spindle parameter across the night for both the participant-mean group analysis and the comparable LMM on individual spindles. Only the incidence of spindles per minute (over the entire set of channels), was not considered using mixed models since, by definition, this a composite measure. Here, we found that incidence decreased over the course of the night from 17.5 spindles per minute to 16.6 (std 1.3 and 2.0; T_8_ = 2.964, p = 0.018); In parallel to this decreased incidence, peak-to-peak amplitude of the spindles also decreased over the night. In contrast to this decrease, the absolute power and its ratio to neighbouring frequencies; the mean spindle duration (early 987.0 ms + − 517.9; late 1120.2 + − 575.7), and the globality of the spindles all increased across the night (early 51.4% + − 19.1; late 57.8% + − 20.3). Moreover, the mean frequency (over the central region of interest), increased from 12.5 to 12.9 Hz.

**Table 1:** Early versus late night comparison between group statistics using participant means and the linear mixed approach on individual spindles for several spindle characteristics.

		Spindle Parameter	duration	amplitude	globality	power	power (ratio)	frontal frequency	posterior frequency	log duration	log amplitude	log power	log power (ratio)
**participant-mean**	**group paired T-test**	Estimated Δ	174.722	−4.403	8.356	0.267	2.007	0.724	0.503	0.180	−0.151	0.043	0.157
		Standard Error	44.972	0.802	1.933	1.141	0.654	0.161	0.213	0.038	0.025	0.114	0.047
		Lilliefors Statistic	0.257	0.192	0.218	0.155	0.160	0.312	0.340	0.214	0.262	0.120	0.161
		t-value	−3.885	5.491	−4.323	−0.234	−3.069	−4.509	−2.358	−4.707	5.999	−0.375	−3.305
		*p-value*	*0.005*	*0.001*	*0.003*	*0.821*	*0.015*	*0.002*	*0.046*	*0.002*	*0.000*	*0.717*	*0.011*
**individual spindles**	**linear mixed model**	Estimated Δ	140.932	−5.139	7.224	1.139	2.659	0.483	0.362	0.130	−0.151	0.051	0.145
		Standard Error	8.044	0.221	0.283	0.208	0.187	0.027	0.026	0.008	0.006	0.012	0.009
		Lilliefors Statistic	0.079	0.111	0.036	0.167	0.150	0.048	0.133	0.032	0.022	0.028	0.029
		LR: Time of Night	304.351	531.280	640.101	30.003	200.271	307.317	195.006	291.753	666.878	16.559	256.858
		*p-value*	*0.000*	*0.000*	*0.000*	*0.000*	*0.000*	*0.000*	*0.000*	*0.000*	*0.000*	*0.000*	*0.000*
		LR: Spindle Type	43.609	139.008	60.537	142.471	194.182	52.164	1357.156	64.187	140.091	180.752	212.561
		*p-value*	*0.000*	*0.000*	*0.000*	*0.000*	*0.000*	*0.000*	*0.000*	*0.000*	*0.000*	*0.000*	*0.000*
		LR: Interaction	3.779	11.337	15.394	30.187	8.687	2.497	20.218	3.628	8.696	29.458	5.658
		*p-value*	*0.151*	*0.003*	*0.000*	*0.000*	*0.013*	*0.287*	*0.000*	*0.163*	*0.013*	*0.000*	*0.059*

The directly measured quantities as well as selected log-transformed versions are displayed. Estimated difference (Δ) indicates the particular model’s expected change in each parameter from an early to late NREM cycle, along with the standard error of that estimate. The Lilliefors statistic is a measure of the deviation from normality of the parameter distribution with values closer to zero being closer to the normal distribution. Likelihood ratio statistics (LR) and associated p-value are presented for the main effects of *time of night* (early versus late), the *spindle type* (frontal, posterior, and co-occurring), as well as the interaction, over-and-above just the model with additional terms. Note that the estimated differences change non-systematically; the distribution of individual spindles are almost always closer to the normal distribution (in the log-transformed cases, drastically lower); and that the mixed model always produces lower p-values.

When calculating the effects of *time-of-night* (early versus later spindles) on these spindle parameters using the LMM approach, all were more statistically significant (lower p-values) than their traditional testing counterparts. Moreover, these would remain significant even after a full-bonferroni correction across the series of tests (which would not be the case for spindle power, power-ratio, or posterior peak frequency in the traditional approach). Lastly, nearly all parameters had distributions closer to the normal distribution (i.e. lower lilliefors test statistic), especially in the log-transformed parameters (see Supplementary Fig. 2 for distribution illustration); indicating that the individual spindle approach is likely less sensitive to outliers and captures the *real* underlying distribution. Finally, the LMM parameter estimates (i.e. the mean value), was robustly different to the overall participant means; indicative that the weighting of distinct total spindle numbers alone likely plays an important role in the analysis.

**Figure 3:**
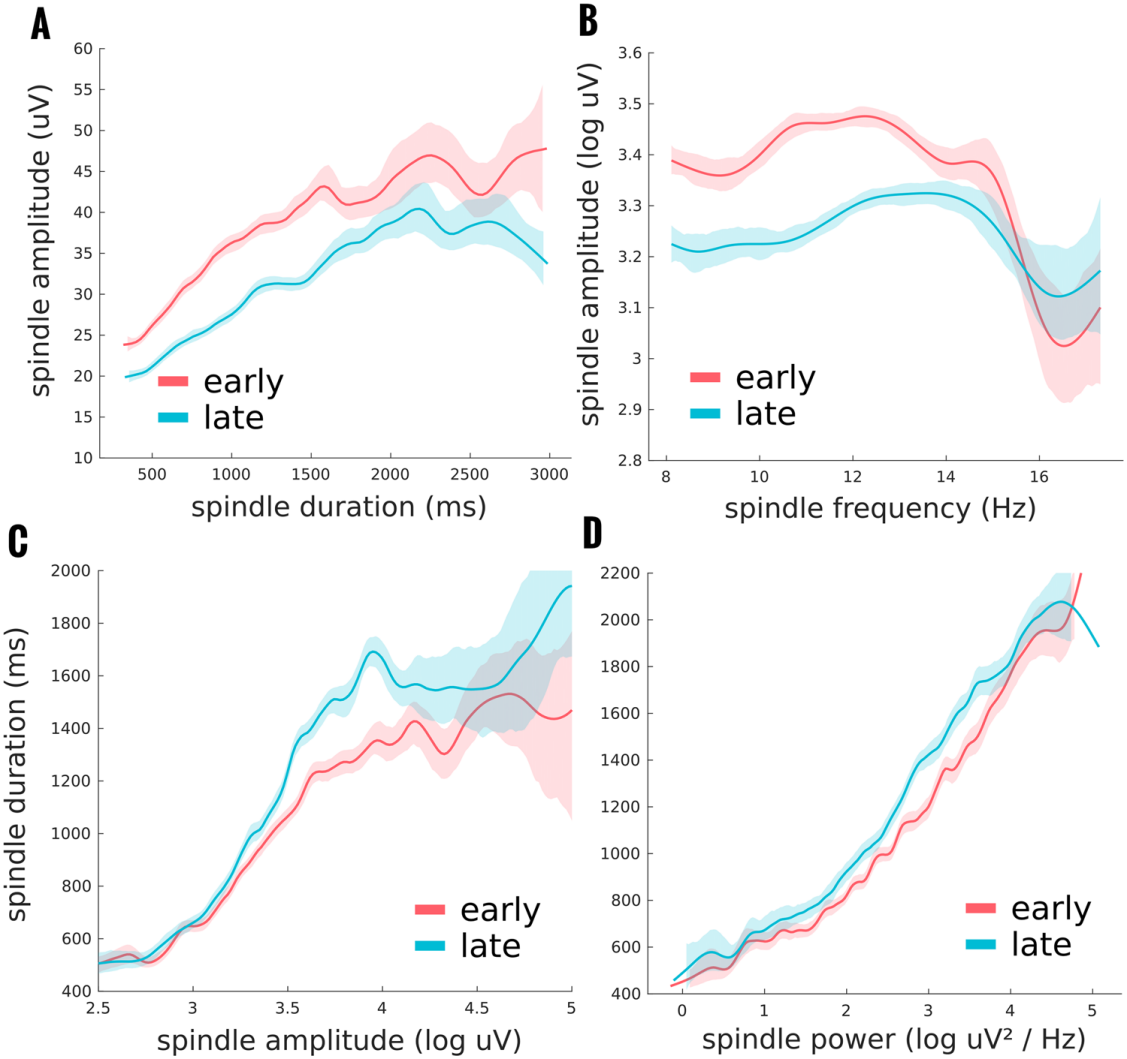
Individual spindle analysis using mixed models showed several interactions of interest. Spindle amplitude, and spindle duration data, see Fig. 2, can also be analysed using a mixed model approach including the other spindle parameters as covariates. In this way, we can examine, not only the condition differences between early and late night spindles, but how these parameters might interact with one another and be modulated by the *time of night*. (**A**) shows how the decrease in spindle amplitude from early to late night is primarily for spindles of shorter duration, and longer duration spindles are more variable in this condition effect. (**B**) Differences in spindle amplitude across the night is also modulated by spindle frequency with only significant differences found for spindles with a power peak in the lower frequency range. (**C**) Spindle duration increases across the night, and this increase depends on the spindle amplitude, with virtually no differences in small peak-to-peak amplitudes which steadily increases until an amplitude plateau when early to late distinctions are variable. (**D**) The effect of *time of night* on spindle duration is slightly modulated by the spectral power of the spindle, with steeper slopes late in the night. Of further note is that while spindle amplitude and duration positively correlate, spindle amplitude reduces across the night, while spindle duration increases.

Given the counter-intuitive finding of a reduction in spindle *amplitude* with an increase in spindle *duration* across the night, we further examined these parameters using the proposed LMM approach with all other spindle parameters as potential covariates (see Fig. 3). Importantly, despite their apparent differential effects across the night, *amplitude* and *duration* significantly correlated at the individual spindle level (r = 0.5728; χ^2^_(1)_ = 4470.1, p < 0.0001). For both *amplitude* and *duration*, stepwise regression found a total 23 terms that constituted the optimal model; including all potential covariates and several interactions. *Time-of-night* had a significant main effect on spindle amplitude (Likelihood ratio of the model with and without the factor: χ^2^_(5)_ = 817.1618, p < 0.0001). Moreover, its effect was significantly modulated by *spindle duration* (χ^2^_(1)_ = 5.9180, p = 0.0150) and the *peak frequency* in the frontal region (χ^2^_(1)_ = 9.3752, p = 0.0022). For *spindle duration*, the dependent variable *time of night* again played a significant role overall (χ^2^_(5)_ = 513.8809, p < 0.0001), which was modulated by *spindle amplitude* as above (χ^2^_(1)_ = 13.4800, p = 0.0002) and *spindle power* (χ^2^_(1)_ = 11.7288, p = 0.0006).

It is important to note that these interactions were significant over and above the other effects in the model, including the other interactions reported. Other interactions with *time of night* were also found to significantly improve the overall model (since they were added by the stepwise regression), yet did not produce effects that were significant over and above others in the model. This does not however imply that these parameters did not have significant effects on the dependent variable directly (e.g. globality: χ2_(1)_ = 1642.9, p < 0.0001), or that would not have significantly interacted if examined in isolation (time of night and globality: χ2_(1)_ = 6.1179, p = 0.0134). Therefore, this approach using all potential covariates could potentially distinguish those with a more direct influence on the dependent variable in question from those that are more like to be intermediary or simply correlated.

Topographical maps were created using paired t-tests between the early and late spindles at the participant level and corrected using the TFCE procedure. Spindle density (in spindles per minute), showed no significant differences in the incidence of spindles classified as predominantly frontal from the first to the last cycle of NREM sleep. However, for posterior spindles there was a significant increase (suprathreshold cluster size of 49 channels), just outside the primary central-posterior region, mostly frontal (peak channel E12; T_8_ = −4.206, p = 0.015). This increase in spindle density can be attributed to changes in the globality of individual spindles, which was shown to significantly increase. Changes in the power ratio at the individual channel level showed significant increases in the central-posterior region, essentially reflecting the hotspot for posterior spindles (67 channels; peak channel E51; T_8_ = −5.028, p = 0.006). Finally, when the shift to higher frequencies was examined across the whole topography, significant increases in peak frequency could be found in a region of 35 frontal electrodes (peak channel E23; T_8_ = −4.371, p = 0.019). Thus, while the increases in power ratio and globility found in the summary measures could generally be attributed to posterior spindles, frontal spindles shifted to faster frequencies.

### Elderly versus paramedian thalamic stroke spindles

**Figure 4:**
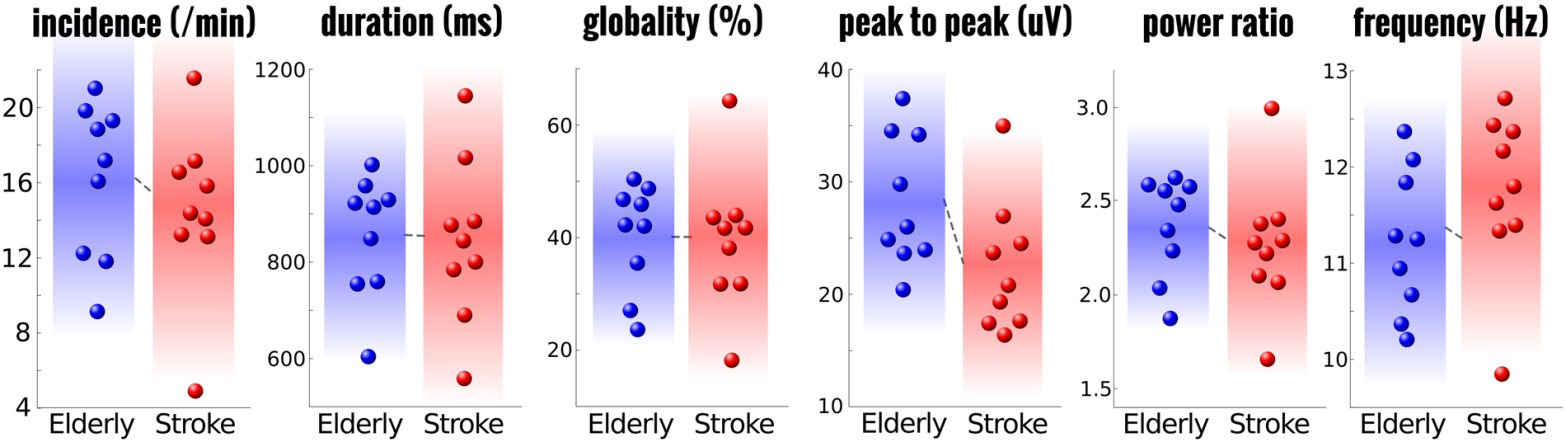
Comparison of spindle characteristics between elderly controls and paramedian thalamic stroke. Individual participant means are shown for each of the selected spindle parameters. Group level statistics on the individual means, without accounting for other crucial covariates such as specific stroke location and the dependence of other spindle parameters, results in no statistical group differences. The background colour indicates the mean value with fade out at 2 standard deviations.

**Figure 5:**
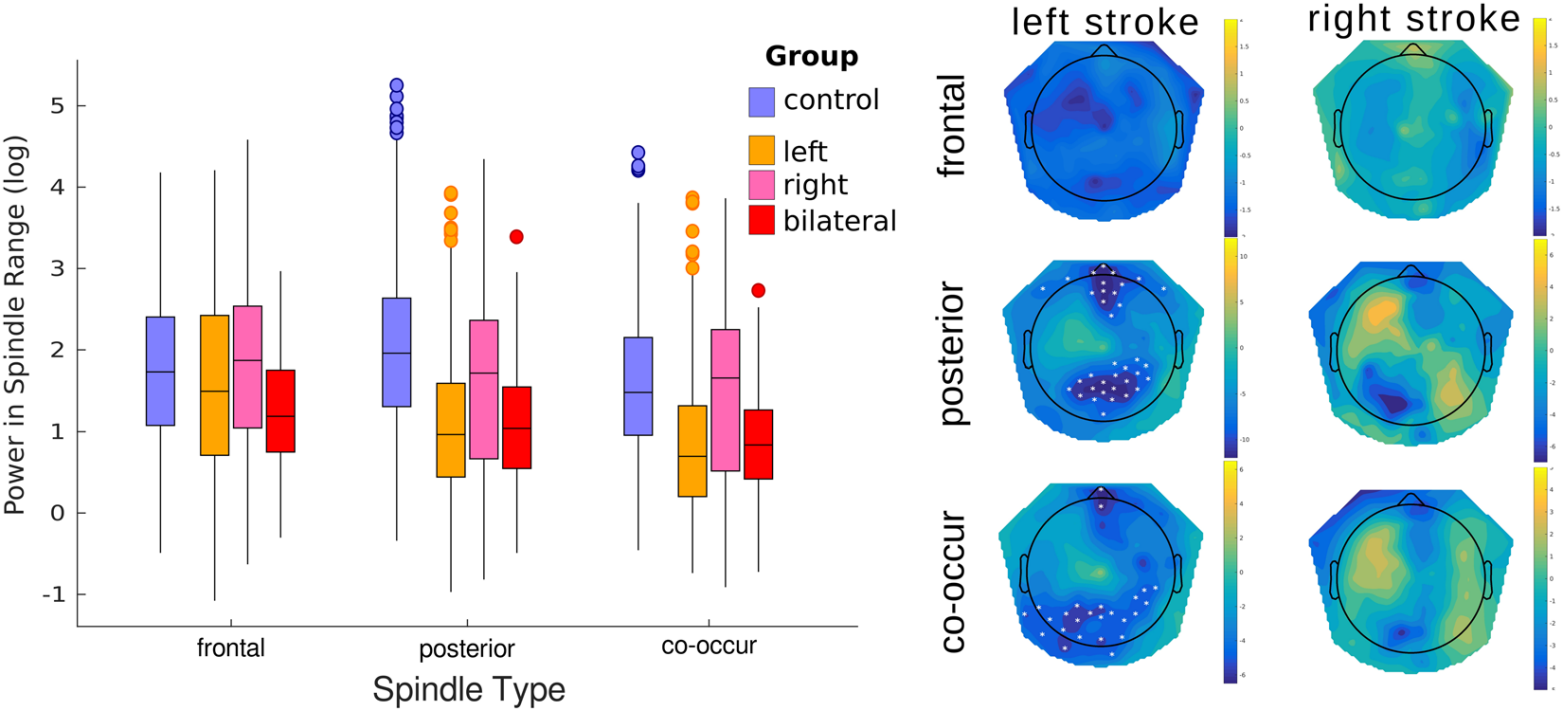
The effect of paramedian thalamic stroke on individual spindle power. Left boxplots show the distribution of spindle power for each of the stroke locations relative to the elderly, age-matched control group for all individual spindles. Linear mixed model analysis showed a significant reduction in power for both the posterior and co-occurring spindles after left sided thalamic damage with no additional loss of power after right damage. Right panel shows the topographic distribution of the calculated parameter estimates from the model at the individual channel level. Channels marked with a white asterisks are considered statistically different after multiple comparisons correction using a permutation approach of the linear mixed model data.

Figure 4 depicts the summary results for the comparison between elderly controls and all paramedian thalamic stroke patients treated as a single group (Left: 3 | Right: 3 | Bilateral: 3). For this analysis, an important question is whether stroke location had a significant effect on spindle power. At the participant level, such an analysis is unfeasible with only 3 participants per group. However using the LMM approach we are examining the properties of individual spindles of which we have over 1000 in each group. Stroke location was specified using 2 factors, *left stroke* and *right stroke*. Healthy controls were assigned ‘false’ on both while bilateral stroke patients were ‘true’ for both factors. In this way, all relations between the patients themselves and controls are specified. In this analysis the log of the spindle power was considered as the primary dependent variable as this corresponds most often to what is measured in the literature. The log of the *duration* and *amplitude* were taken so that their distribution was approximately normal (see Supplementary Fig. 2). Collinearity was removed from the key predictors by taking the residuals as above. Fig. 5 summarises the findings from the LMM approach. As before, several models were examined using the stepwise algorithm with additional interactions specified and tested using the likelihood ratio test between models. The best model consisted of the interactions between *spindle type* and both *left* and *right stroke* (but not the three way interaction), as well as the explanatory covariables of *duration, globality, peak-to-peak amplitude*, and both the *peak frequency* at the parietal and frontal canonical regions (9 total predictive variables).

This model revealed a significant interaction between *spindle type* and *left stroke (*χ^2^_(2)_ = 102.0365, p < 0.0001), but not *spindle type* and *right stroke* (χ^2^_(1)_ = 2.5857, p = 0.2745). The interaction was driven by significantly reduced spindle power only for posterior and co-occurring spindles (χ^2^_(3)_ = 13.8553, p = 0.0031 and χ^2^_(3)_ = 12.2274, p = 0.0066 respectively), in patients with a left thalamic stroke (including bilateral stroke). No group differences were found for frontal spindles (χ^2^_(3)_ = 3.5356, p = 0.3162). This model consisted of a total of 70 terms, yet the relatively simpler model (25 terms) derived from the stepwise approach alone (limited to two-way interactions), would have resulted in the same key significant and non-significant terms with only marginal differences.

Given the high locality of spindles, and the finding that mainly left sided stroke appears to significantly affect spindle power, it is interesting to examine the topographical distribution of power in these groups using LMM. The results generally reflect those of the summary measure reported above. For frontal spindles, no channels were found to show a significant difference compared to the elderly controls. However, two large clusters of differences were found for the effect of *left stroke* indicative of a reduction in power for parietal spindles. The larger, parietal cluster (21 channels) of *left stroke* effects had its peak at the Pz electrode (T = 12.316, p < 0.001). The smaller, frontal cluster showed a peak at E15 (T = 14.020, p = 0.006). For those spindles classified as co-occurring there was a clear reduction in spindle power in far posterior channels with a slight left bias (23 total channels; peak channel = E66; T = 5.723, p = 0.019). Two further channels in the frontal region also showed a significant reduction (peak channel = E17; T = 5.687, p = 0.039). No channels showed differences for the *right stroke* parameter (peak channel E105; T = −1.404, p = 0.473).

While we found that the topographic peak loss of power after left-sided stroke was located at the central parietal channel, we performed an additional analysis to explore whether parietal channels in the left or right hemisphere were particularly more affected. We extracted the mean power values of 3 parietal channels on either side of the peak effect, and ran a single LMM including *channel side* as a fixed effect and *spindle number* as a random effect (to account for the expected correlations between power in each hemisphere at the individual spindle level). Contrary to the topographic appearance of symmetry, we did find a significant three-way interaction between *left stroke, right stroke* and *channel side* (χ^2^_(1)_ = 6.4376, p = 0.0112). While the overall pattern of a significant reduction in power was confirmed from the summary analysis above, in patients with only a left-sided stroke the spindle power in the right region of interest was slightly more reduced. In contrast, for bilateral stroke patients, the channels on the left side were more reduced. Compared to the overall reduction of spindle power of 15.5% after left-sided stroke found in the initial analysis, the modulation of *channel side* was a modest 3.8%. Interestingly, this analysis also found an interaction between *right stroke* and *spindle globality* (χ^2^_(1)_ = 4.6062, p = 0.0319). The interaction indicated there was a significant reduction in spindle power in both regions only for those with low globality (aka, more local spindles) after *right stroke*.

## Discussion

Our primary aim was to demonstrate how spindle analysis could be adjusted and expanded into hd-EEG recordings. We showed how this approach can produce novel and interesting findings in distinct groups of young adults and brain damaged patients. Moreover, we highlighted how distinct parameters can be derived from the detection and how those related to each other. We introduced the LMM approach to statistically analyse spindles at the individual level and showed how this changes parameter estimates and increases the statistical significance of the results compared to using participant means. Importantly, this allowed for larger models which included several covariates to *account* for variability in the data; instead of treating this as measurement error. The LMM approach was then extended to the topographical comparisons involving thalamic stroke which revealed local changes in spindle power.

One of the first striking findings is the sheer number of detected spindles per minute. Previous studies have reported comparatively low numbers around 2–7 spindles per minute^36,37^. We found spindle densities with a range from 13–20 (mean of 18 in healthy controls; see Fig. 1). It is possible that our method leans more toward sensitivity, with a corresponding trade-off of more false positives. Yet this is unlikely to fully account for our results for multiple reasons. Firstly, when examining the topographical distribution of spindles per minute (see Fig. 2), we see that no single channel has a density of more than 6.5. Thus, if spindles are only detected at a single channel (the case in many studies), the incidence here would be comparable. Similarly, most studies tend to use a central and often lateral derivation (e.g. C3/C4), when detecting spindles^15,38^. We find spindles to be primarily a midline phenomenon, with densities in those lateral channels typically around 3–4 spindles per minute. Not only do spindles occur less regularly in those channels, but in our analysis, a spindle that has its peak elsewhere could still be detected; while it may not pass detection criteria when examining those lateral channels alone. Lastly, technically an oscillation is only labeled as a spindle if longer than 500ms^39^, however, as Warby *et al*.^15^ already noted, algorithm performance dramatically improves if this criteria is lowered to 300 ms as was done here. This change in classic criteria accounted for a mean of 16.3% (+−5.2%) of the spindles found in our young control group. Thus also contributing to the increase in spindle incidence reported here. Furthermore, there is a growing body of literature to suggest that spindle incidence is actually much higher than classic studies would suggest. Recent research using multimodal measurement techniques (intracranial electrodes^3,26,40^; MEG^41–44^; or fMRI^12,45^) show a generally low level of synchrony between these and scalp recordings, suggestive of a high level of locality in spindles. This would lead to underreporting of spindle activity at the scalp level; especially in the cases of spatial undersampling.

Spectral power differences used in previous studies have usually been attributed to changes to either spindle incidence or amplitude. However, in the early versus late night comparison individual spindle power increased over the course of the night, yet the usual suspects, incidence and amplitude, actually decreased. This left spindle duration and globality as the positive correlates to power. Three important messages come from this finding. Firstly, spindle properties are differentially affected across the night. While we cannot disentangle the effect of circadian rhythm and the expected reduction in sleep pressure across the night, both incidence and amplitude track the homeostatic decrease commonly observed in slow-wave activity, while an opposite increase in duration, peak frequency and spread over the cortex was observed^25,46^. The second observation is that using power measures alone may miss changes to spindles if these, more directly related factors, are sufficiently balanced. Therefore, if only spectral analysis is performed, speculation about any specific spindle parameters which underlie these changes could be misleading. Lastly, their relative independence suggests that distinct structures, networks, or patterns of activity are responsible for each phenomenon as opposed to a single structure (e.g. reticular thalamic nucleus^47^).

In the majority of the literature on spindles, the distinction has been made between fast and slow spindles^48,49^, whereas here we categorised each spindle by its topographic maximum. The primary motivation for this shift here is that in much of the previous literature, the distribution of frequencies has been relatively normal (e.g. see Fig. 2b in^15^; Fig. 5 in^50^), and the classification has relied on an arbitrary split of this distribution. When sufficient channels are recorded, and the topography of individual spindles can be examined (as the case here and shown in Fig. 1), there is a clearer binary separation of spindles by their topography. While both these classifications are largely overlapping, they are not completely redundant since all the models tested showed significant impacts when information about spindle location and peak frequency was provided. Moreover, as Fig. 3 indicates, certain spindle parameters are only modulated across the night in specific frequency bands. Distinct spindle pathways, is a more physiologically plausible and parsimonious dichotomy between spindle types. This idea is supported by the recent findings using intracranial EEG to detect spindles which suggest that the specific spindle characteristics, especially their fundamental frequency, depend mostly on the cortical location where they are expressed^13^. However, further work, particularly in perturbational approaches which cause or modulate spindle activity, will illuminate this issue^51^.

Our comparison between stroke patients with a paramedian thalamic stroke revealed that essentially only damage to the left side of the thalamus resulted in a significant reduction in individual spindle power. Moreover, patients with bilateral thalamic damage showed the same pattern as those with left side damage alone. The lateralised effects of thalamic damage have been previously documented by Hermann *et al*.^52^. They found that patients with left or bilateral thalamic damage had a significantly worse performance in memory-related tasks than those with exclusive damage to the right; even a year after the stroke. They suggested that this poorer outcome may have to do with the lateralisation of hippocampal loops role in language, memory and error monitoring. While this may indeed be the correct interpretation, our results tentatively suggest that sleep spindles are directly impaired, which may cause poorer consolidation of sleep with only subsequent effects on memory^53^. It should be noted that despite the laterality in pathology, the sleep spindles themselves were only mildly laterally affected. This finding is consistent with previous literature exploring spindles after unilateral thalamic stroke^10^, and unlike findings after hemispheric cortical stroke^44,53^. While our results are consistent with findings relating left thalamic damage to memory impairment, we note that bilateral damage tends to produce more severe deficits compared to unilateral damage alone for broader clinical measures such as frontal deficits, hypersomnia, or visual impairments^9,54^.

Future work should take advantage of recent advances in structural and functional imaging techniques, as well as lesion segmentation analysis, to better characterise lesion locations and extent as these will undoubtedly lead to improved specificity within the LMM approach^55–57^. In particular, research specifically comparing laterality of lesions is scarce. One should also keep in mind that while the statistical approach is valid for the current dataset, generalisation to a greater population of patients is nevertheless limited by the small number of total patients examined and future studies should aim to measure more patients to verify the effects. More detailed analysis of the stroke lesion from neuroimaging may find certain biases in lesion sizes, including structural and functional connectivity, that better account for the lateralisation found here. As such we are hesitant to make definitive conclusions about the changes one can expect to spindles in a future group of patients. Given the limited sample sizes in our analyses and that of future studies, especially in the clinical domain, these results may serve as the empirical basis for power analyses using simulations on the linear mixed models^58^. We emphasise that the main purpose of the current investigation was to present the tools, in both detection and analysis, that could be used in these future studies to maximise the potential information within the datasets.

## Electronic supplementary material

Supplementary Information
