# Supplementary material for: Individual spindle detection and analysis in high-density recordings across the night and in thalamic stroke
Source: Sci Rep. 2018 Dec 14;8:17885. doi: 10.1038/s41598-018-36327-x (PMC6294746; doi:10.1038/s41598-018-36327-x)

Individual spindle detection and analysis in high-density recordings across the night and in thalamic stroke

Mensen^1,2^, A., Poryazova^3^, R., Huber^4^, R., Bassetti^1^, C.L.

1. Department of Neurology, University Hospital (Inselspital) and University of Bern, Bern, Switzerland

2. Giga-Consciousness, Coma Science Group, University and University Hospital of Liege, Belgium

3. Department of Neurology, University Hospital Zurich, Zurich, Switzerland

4. Child Development Center, University Children's Hospital Zurich, Zurich, Switzerland.

#

Supplemental Information


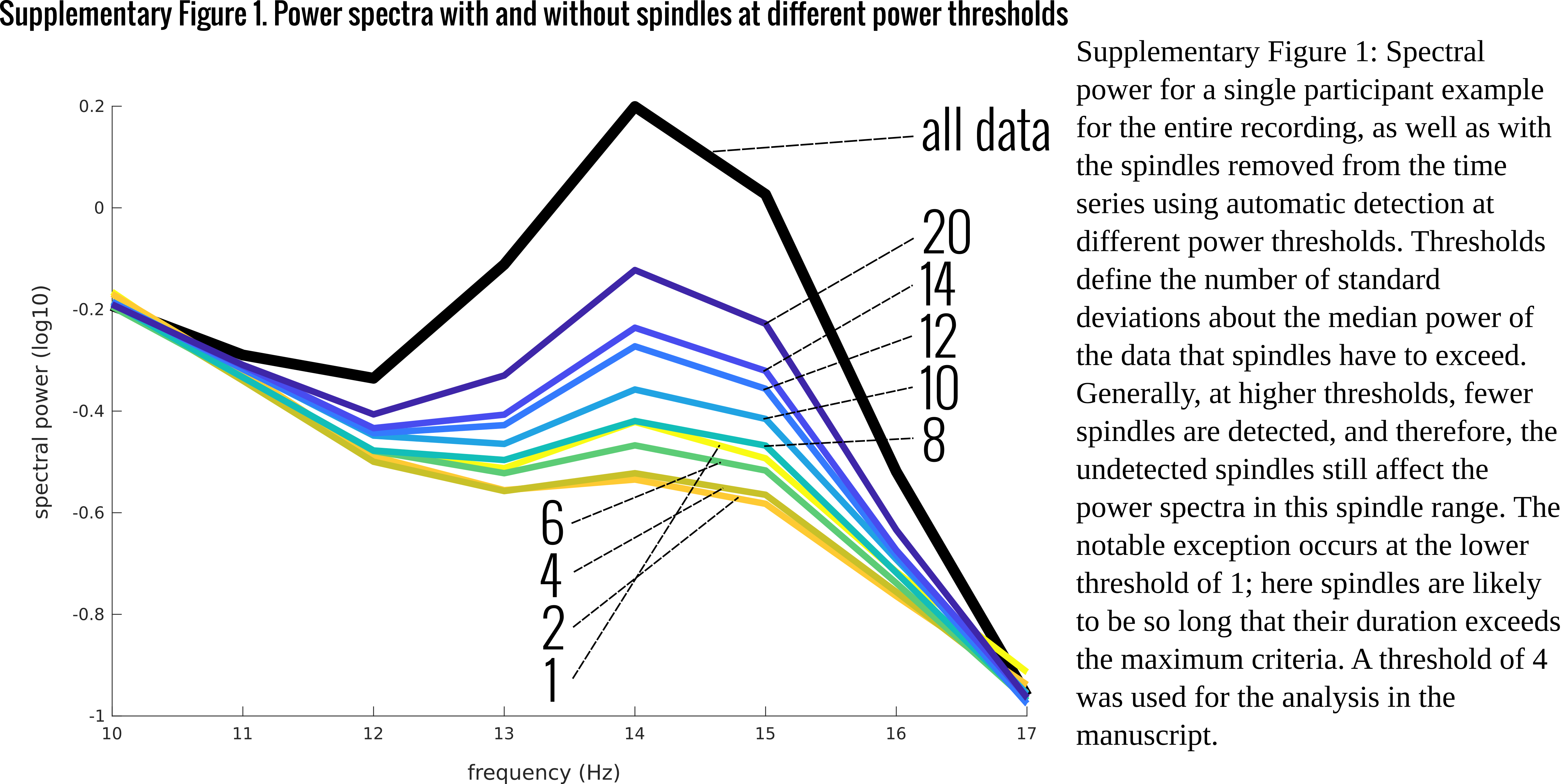


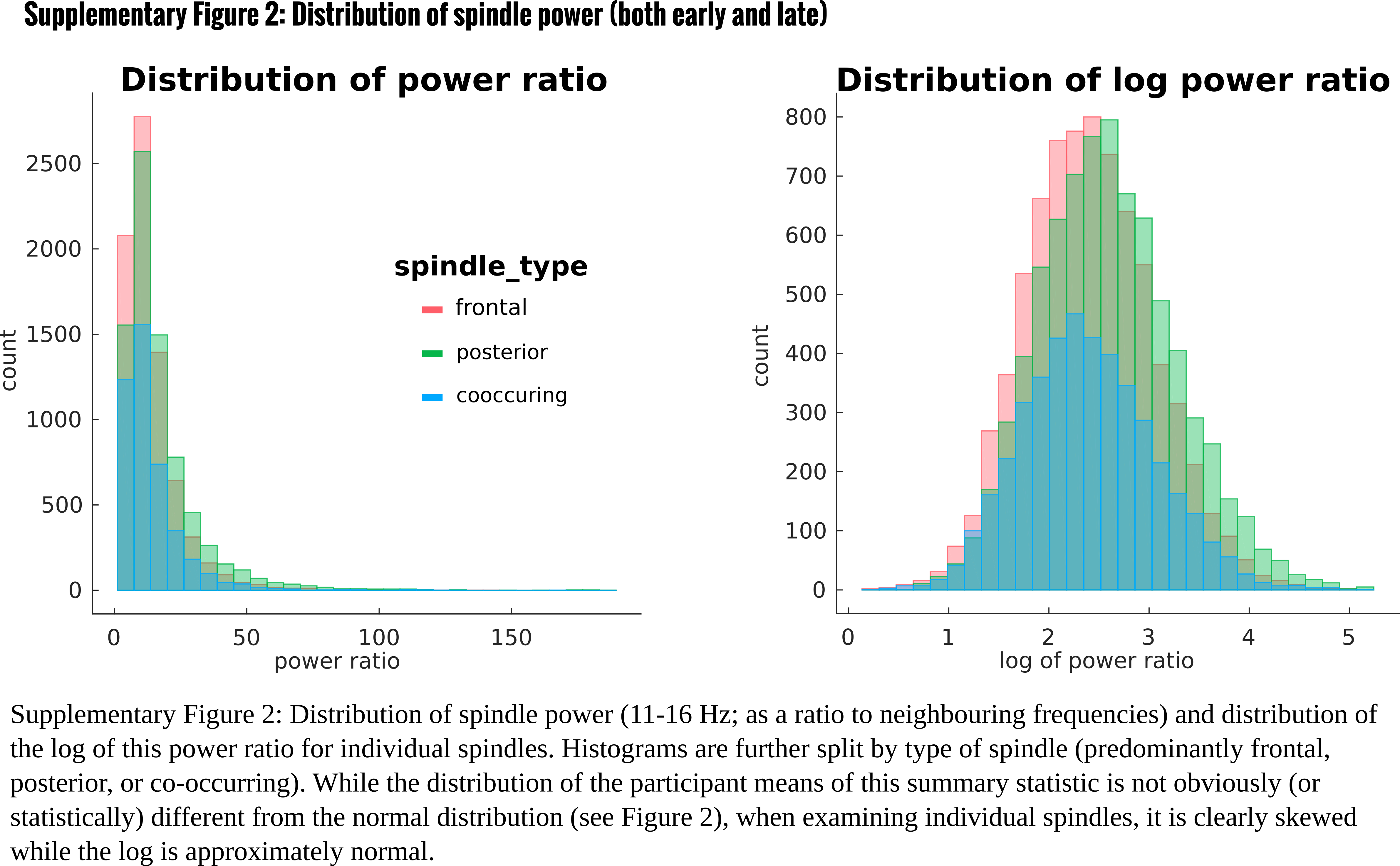


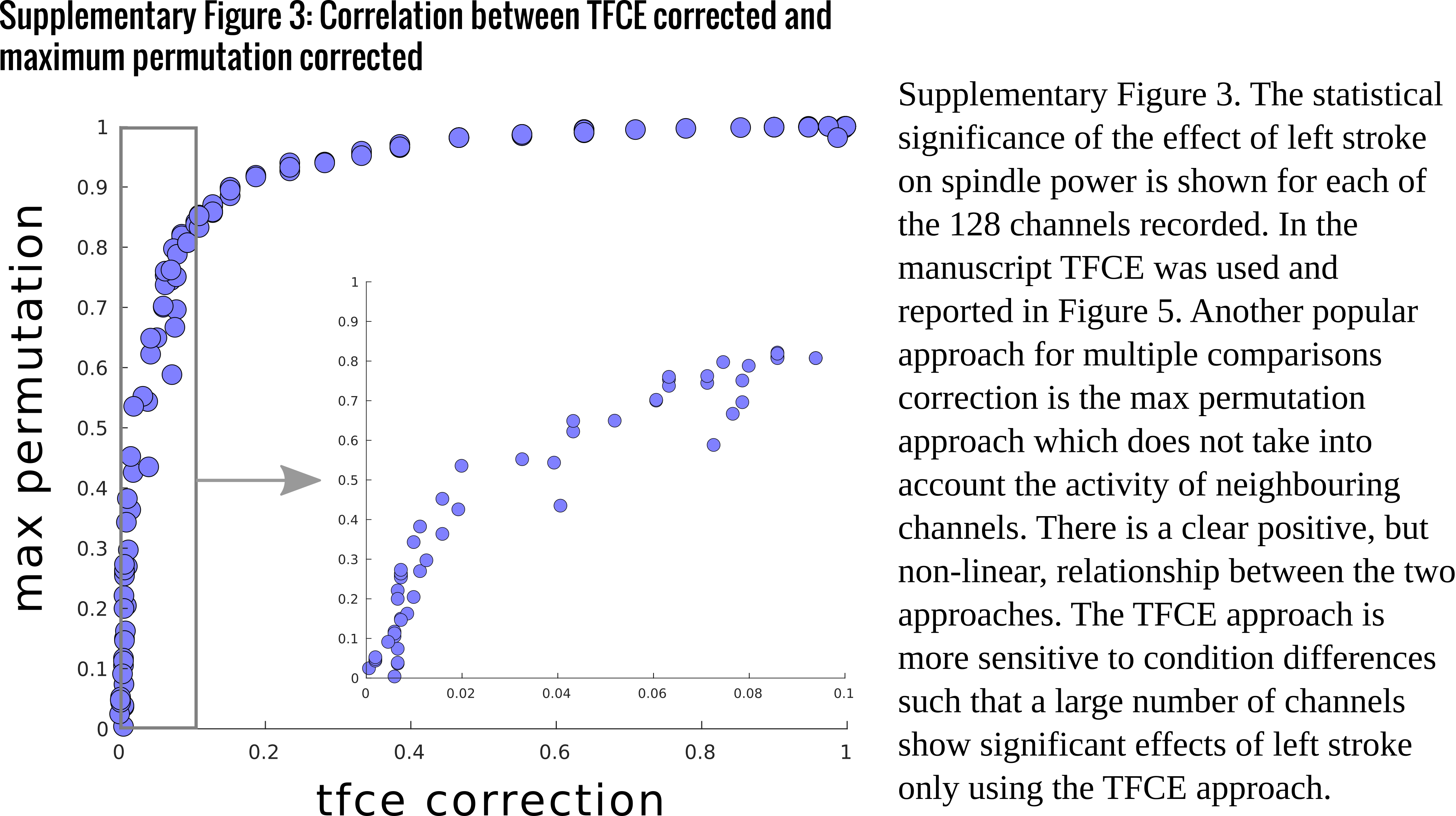

Supplement: Supplementary file 1 — Supplementary Information [file 41598_2018_36327_MOESM1_ESM.docx]
